# Bruxism in Children and Adolescents with Down Syndrome: A Comprehensive Review

**DOI:** 10.3390/medicina57030224

**Published:** 2021-03-01

**Authors:** Elisa Luconi, Lucrezia Togni, Marco Mascitti, Andrea Tesei, Alessandra Nori, Alberta Barlattani, Maurizio Procaccini, Andrea Santarelli

**Affiliations:** 1 Department of Surgical and Special Odontostomatology, Umberto I General Hospital, 60126 Ancona, Italy; eliluconi@gmail.com (E.L.); andrea.tesei@outlook.it (A.T.); alessandra.nori@ospedaliriuniti.marche.it (A.N.); 2 Department of Clinical Specialistic and Dental Sciences, Marche Polytechnic University, 60126 Ancona, Italy; togni.lucrezia@gmail.com (L.T.); m.procaccini@staff.univpm.it (M.P.); andrea.santarelli@staff.univpm.it (A.S.); 3 Department of Clinical Sciences and Translational Medicine, Tor Vergata University, 00133 Rome, Italy; barlattanialberta@gmail.com; 4 Dentistry Clinic, National Institute of Health and Science of Aging, IRCCS INRCA, 60124 Ancona, Italy

**Keywords:** sleep bruxism, awake bruxism, bruxism, Down syndrome, children, adolescents, obstructive sleep apnea

## Abstract

The role of bruxism in children and adolescents with Down syndrome, the most often diagnosed congenital syndrome, is still unclear. Therefore, this study aims to conduct a narrative review of the literature about bruxism in children and adolescents with Down syndrome to identify the prevalence, risk factors, and possible treatments of this disorder. Although an accurate estimate of its prevalence could not be inferred, it appears that bruxism is more prevalent in Down syndrome individuals rather than in the general pediatric population. No gender difference was observed, but a reduction in its prevalence was described with increasing age (around 12 years). The variability in the diagnostic techniques contributed to the heterogeneity of the literature data. Clinicopathological features of Down syndrome, such as muscle spasticity, oral breathing, and a predisposition to obstructive sleep apnea, may suggest a higher prevalence of bruxism in this patient group. Finally, given the paucity of studies on the management of bruxism in this population, it was not possible to outline a standard protocol for the non-invasive treatment of cases in which an observational approach is not sufficient.

## 1. Introduction

Bruxism is defined as “a repetitive jaw-muscle activity that is characterized by clenching or grinding of the teeth and/or by bracing or thrusting of the mandible,” and is distinguished into sleep bruxism (SB) or awake bruxism (AB), depending on its circadian phenotype [1]. SB is defined as a masticatory muscle activity during sleep that is characterized as rhythmic (phasic) or non-rhythmic (tonic), while AB is a masticatory muscle activity during wakefulness, characterized by repetitive or sustained tooth contact and/or by bracing or thrusting of the mandible [2]. In both definitions, it has been specified that bruxism “is not a movement disorder in otherwise healthy individuals”, to underline that whilst in most persons bruxism is not necessarily pathologic, it may represent a sign of a disorder in others, such as individuals with a rapid eye movement behavior disorder, obstructive sleep apnea (OSA), epilepsy, Down syndrome (DS), and other conditions that require full attention by a responsible clinician [3].

The etiology of bruxism has always been surrounded by controversies, though the current literature supports the view that bruxism is mainly regulated centrally, not peripherally (i.e., not caused by anatomical factors like occlusal interferences, as previously supposed) [3,4,5,6,7]. This suggests that psychological factors (such as emotional stress [8]), medications [9], central nervous system (CNS) disturbances [10], OSA [11], genetic predisposition [12], caffeine, alcohol, tobacco, and drug abuse [13] may have more influence in bruxing activity rather than morphological factors.

SB is a very common finding in children. Systematic reviews have reported a highly variable prevalence, ranging from 6% to 50% [14], depending on the diagnostic criteria to assess it and the different age groups investigated. No gender difference has been observed, and a reduction in its prevalence has been described with increasing age (around 9 to 10 years) [15]. Possible AB, on the other hand, is a rarer condition. Studies have revealed lower prevalence rates compared to those of SB (e.g., 8.7% versus 14.8% [16] and 12.4% versus 15.0% [17], respectively), except for two Israeli surveys in which an inverse trend was observed (19.2% versus 9.2% [18] and 34.5% versus 14.8% [19], respectively).

While bruxism in children is a self-limiting phenomenon, not associated with significant symptoms [20], its role in individuals with DS is less clear. DS is the most often diagnosed congenital syndrome (prevalence, 1/792 live births [21]) related to the presence of a supernumerary chromosome 21. It affects multiple bodily systems, particularly the musculoskeletal, neurological, and cardiovascular systems. Its phenotype is associated with a broad spectrum of cognitive and physiological disorders, which include obesity, hypotonia, relative macroglossia, and delays in motor and neurological development. Moreover, considering that one of the most typical clinical features of DS is the predisposition for sleep disorders, such as obstructive sleep apnea syndrome (OSAS) [22] and its associated parasomnias, it seems worthwhile to deepen the knowledge about it. The aim of this narrative review is to investigate the prevalence, the risk factors, and the management of bruxism in children and adolescents with DS, to raise awareness of this disorder.

## 2. Epidemiology

In the literature, the studies that have investigated the prevalence of bruxism in children with DS are few and not easy to compare. Prevalence data were extracted from 15 cross-sectional between 1973 and 2020.

The highest values were reported by Gullikson [23], who described a prevalence of bruxism of 79% in 28 American children with DS between 3–10 years old; Alarí [24], 70% in 21 Spanish children (age range, 6 to 7 years); Lamma and Cocchi [25], 60.61% in 33 Italian children; and Bell et al. [26], who compared the frequency of severe tooth wear in 49 DS children with 49 non-DS controls (67.4% versus 34.7%). Further, the questionnaire submitted to the parents of children with DS (and completed by 71.4% of them) showed that 11.4% of these children grind their teeth during sleep, 8.6% presented AB, while 11.4% were both diurnal and nocturnal grinders. Rodríguez Peinado et al. [27] compared the prevalence of bruxism among 15 Spanish children with DS and 13 with cerebral palsy (CP), aged 6 to 20 years, in both groups. Even if it is not possible to extrapolate the exact percentage of prevalence in the group with DS, it was discreetly higher than in the group with CP (30.8%).

Borea et al. [28] reported more moderate values: 45% in 37 Italian children (mean age 12 years old); and Ashworth et al. [29] likewise, 45% in 22 children (age range, 6 to 12 years). Carter et al. [30], in a study carried out in the United Kingdom with 58 children/adolescents (aged 4–18 years), found that 45% of the sample presented bruxism at least twice a week; while Breslin et al. [31], in a similar survey conducted on 35 patients (age range, 7 to 18 years), described a prevalence of 34%. López-Pérez et al. [32] sought to determine the prevalence of bruxism in a Mexican community of 57 children with DS, ranging from 3 to 15 years old. The overall prevalence was 42%, with no statistically significant associations between bruxism and age and sex. However, the authors showed a marked decrease in this behavior starting from the age of 12 years old. A parallel trend was described by Ruy Carneiro et al. [33], with a prevalence of 51.8% among 112 children (aged 5–16 years), and a lowering of the value since the age of 13. Maris et al. [34] also observed a negative association between parasomnias and age (*p* = 0.014 (B = −0.333)), and an overall prevalence of SB of 31.5%, in a sample of 54 Flemish children (age range, 5.4–11.6 years). 

Only Hernández et al. [35] and Miamoto et al. [36], reported lower rates: the former, 18% in 61 Mexican children (range 6 to 14 years), and the latter 23% in 60 Brazilian children. Similarly, Areias et al. [37] found a prevalence of bruxism of 23% in 45 Portuguese children with DS (aged 6–20 years). That value was high when compared with the prevalence in their typically developing siblings (23% versus 2%). This difference was statistically significant (*p* = 0.004).

A high variability has emerged for the overall prevalence rates, ranging from 18% up to 79%. The reason for such discordant data may lie in the fact that each study differs in the age group investigated and that, in some research, the sample of children examined included a wide range of ages. Furthermore, the majority of surveys did not discriminate between SB and AB. Notwithstanding the above limits, some interesting deductions can be gathered from the data obtained. A recent systematic review of the literature regarding SB in children highlighted an inverse relationship between the prevalence of bruxism and age (i.e., the prevalence of bruxism decreased with age in all the surveys) [15]. This trend appears to be common even in children with DS [32,33,34], where the highest prevalence is found in the age group 6–12 years, followed by a tendency for a progressive decrease in this behavior. No gender-related significative differences have been observed, as in the typically developing children [15]. Regarding AB, it has been hypothesized that it does not vary with age [38]. Finally, even though it is not possible to infer an accurate estimate of the prevalence of bruxism in children with DS, it seems that this disorder is more prevalent in these individuals than in the general pediatric population, whose reported prevalence rates range from 5.9% to 49.6% [14]. The literature prevalence data of SB in Down syndrome patients have been summarized in Table 1.

## 3. Diagnosis

The variability in the diagnostic techniques used also contributes to the lack of homogeneity of the prevalence values reported above. Information about the diagnosis of bruxism in children with DS were extracted from 9 studies, published between 1990 and 2018.

According to the updated international consensus criterion, bruxism is classified into three categories: possible SB/AB (based on caregiver report or self-report); probable SB/AB (positive clinical inspection, with or without a positive self-report or caregiver report); and definitive SB/AB (positive instrumental assessment, with or without a positive self-report or caregiver report and/or a positive clinical inspection) [2]. In almost all surveys, bruxism was evaluated through non-instrumental methods, which included a questionnaire completed by the patient’s relatives or caregivers, intraoral examination, and/or analysis of dental study casts. Four research teams administered the 33-item Child Sleep Habits Questionnaire (CSHQ) [39], a screening instrument for school-aged children based on common clinical symptom presentations of prevalent sleep disorders. Parents are asked to score sleep behaviors basing them on a “typical” recent week. Each item is scored on a 3-point scale depending on its frequency: usually (5 to 7 times/week), sometimes (2 to 4 times/week), or rarely (0 to 1 week). The CSHQ allows for a total score (>41 in the clinical range, indicating an underlying sleep problem) and 8 subscales or domains: sleep resistance, sleep onset delay, sleep anxiety, sleep duration, nocturnal awakenings, parasomnias, sleep-disordered breathing, and daytime sleepiness. The cut-off is set at a score of 41 from the point of intersection of sensitivity and specificity, 0.80 and 0.72, respectively, and is considered to be the best diagnostic confidence [39]. In the remaining studies, a questionnaire not otherwise specified was used to investigate sleep bruxism. Thus, the first problem encountered is the noncomparability of the scores obtained from these tests. Secondly, intra-oral and/or dental casts examination, looking for wear facets, may provide biased results, tending to underestimate or overestimate the incidence. In fact, although tooth wear can be objectively measured, it may not be indicative of the actual level of bruxism, because individuals who have bruxed in the past may present with dental facets, while individuals who have recently started bruxism may not yet have signs of tooth wear [40].

The gold standard technique for the diagnosis of SB is polysomnography (PSG) [41]: a detailed analysis in a sleep laboratory of physiological and neural activity during sleep. The negative aspects of this examination include costs, the long waiting list at public hospitals, and that sleep is not monitored in a familiar environment, becoming a distressful element for children with developmental disorders. Since it is such a demanding exam, PSG is usually reserved for the OSA screen. Recently, actigraphy has been proposed as a reliable and valid alternative that can be used to continuously assess activity levels in a home setting over an extended period. It is inexpensive, nonintrusive (requiring only that an actigraph watch be worn), and it is well tolerated by children with DS [42]. Actigraph watches are devices the size of a standard wristwatch, capable of recording data while a highly sensitive accelerator records movement. They have been used to evaluate sleep/wake adaptation and circadian cycles [43]. Actigraphy demonstrated convergent validity with PSG when measuring a child with DS’s total amount of sleep time, total wake time after sleep onset, and sleep period efficiency. In contrast, actigraph watches showed poor correlations with PSG when measuring the total time in bed and total wake episodes [42]. Although actigraphy is sensitive to sleep-related respiratory disturbances, it is not suitable for assessing such disorders [44]. However, this diagnostic tool may prove useful in the diagnosis of SB, because evidence exists that this behavior may represent a sleep disorder that appears concurrently with the transient arousal response [45]. Only one of the studies previously reported [28] used actigraphy, supported by the administration of the CSHQ to parents.

Despite the fact that non-instrumental and instrumental approaches to assessing bruxism may have a poor concordance, self-reporting of SB or AB continues to be the primary tool in bruxism research and clinical practice [42] because it is easier to perform and provides large amounts of data, especially for large-scale surveys.

A comparison of these studies reveals the lack of common diagnostic tools; a starting point could be the identification (e.g., the CHSQ, which has demonstrated validity in pediatric populations characterized by intellectual and developmental disabilities [46]) of a universally accepted questionnaire to assess SB in children with DS.

## 4. Etiology and Risk Factors

The investigation of etiology and risk factors for bruxism in children with DS was conducted in 5 studies, published between 1974 and 2020. In the professional literature, the correlation between emotional stress and SB in the pediatric population has been well and consistently documented [47,48,49], but there are other relevant factors as well. Heavy exposure to second-hand smoke, sleep disturbances, functional and parafunctional habits (such as primary canine wear, clenching teeth during the day, and biting objects), behavioral abnormalities, and psycho-social factors (in particular, high levels of responsibility) were found to be the risk factors for bruxism with the highest odds ratios [48,49].

All of these risk factors may be present in children with DS, but other elements have been investigated that may explain the increased prevalence of bruxism in this population. Firstly, these individuals are characterized by the presence of spastic muscles that are more prone to contraction and require more effort to relax [36]. The physiological pattern of alternation between jaw-opening muscles and jaw-closing muscles is generated by the masticatory central pattern generator (CPG), located in the brainstem. In bruxism, this central regulatory mechanism is disrupted, and co-activation of both muscle groups is observed [50]. Therefore, muscle spasticity could play an important role in the etiology of bruxism in subjects with DS [51].

Ruy Carneiro et al. [33] observed a statistically significant association (*p* = 0.022) between oral breathing and reported bruxism in children/adolescents with DS. This is related to the health complications characteristic of this syndrome, such as adenoids and palatine tonsils hypertrophy, which can result in respiratory distress and sleep disruption. Sleep interferences can lead to involuntary muscle contraction of facial muscles, causing SB [52]. DS patients also have a considerable number of oral problems, such as missing or impacted teeth, posterior crossbite, open bite, and other dental anomalies [53]. The survey conducted by Miamoto et al. [36] revealed that DS children with sucking habits (*p* = 0.007) were four times more likely to exhibit SB, while the presence of posterior crossbite (*p* = 0.017) increased the risk of SB by approximately threefold. Also, in controls, posterior crossbite was associated with SB. This finding does not corroborate with the results of subsequent studies [54,55] that have not found any association linking between SB and posterior crossbite in typically developing children. Although this malocclusion may generate an increased propensity for mandibular deviation during the physiological activities of the teeth [36], the notion that peripheral inputs, such as incorrect occlusal contacts, could trigger the mechanism of bruxism must be abandoned.

Deepening into sleep disorders, it is impossible not to mention OSA, whose relationship to SB has been unambiguously clarified [11,56]. This comorbidity associated with DS negatively affects the quality and restorative value of sleep through its “fragmentation”. It is a complication present in 50% or more of children with DS [57], compared with 2–3% in the general pediatric population [58], precisely because OSA results from a combination of factors (e.g., soft tissue and craniofacial abnormalities, fatty deposition in the neck, hypotonia, and relative macroglossia) that are the hallmarks of DS [59]. 

Considering bruxism from a different perspective, further justification for its higher prevalence can be found. Indeed, children with DS exhibit repetitive behaviors (such as unusual routines, stereotypies, and rituals) more frequently than the general pediatric population. Repetitive behavior is an umbrella term used to describe behaviors characterized by frequency, repetition, inappropriateness, and invariance [60]. These behaviors can be distinguished into motor or “lower-order” repetitive behaviors (e.g., stereotypic movements and self-injury) and more complex or “higher-order” repetitive behaviors (e.g., ordering, checking, washing). Lower-order behaviors may be coping reactions used to modulate stress or arousal [61], and AB fits the criteria for inclusion into this group. The role of the level of cognitive impairment still remains unclear. Whilst Lindqvist and Heijbel [10] have shown that the degree of mental retardation is a major determinant in the pathogenesis of abnormal tooth wear, these findings have not been corroborated by subsequent studies conducted by Borea et al. [28], Miamoto et al. [36], and López-Pérez et al. [32]. Finally, López-Pérez et al. discovered that there was a statistically significant difference across the different types of Trisomy 21; in particular, genetic mosaicism is the most frequently associated with bruxism [32].

## 5. Consequences

Bruxism does not have to be exclusively associated with negative consequences. As previously stated, it is not a movement disorder in otherwise healthy individuals. Moreover, in some circumstances, it can even be considered a positive behavior. In the only study that examined the consequences of this clinical condition, bruxism was identified as one of the protective factors for the lower incidence of caries in children with DS, compared with their typically developing siblings [37]. In fact, tooth friction seems to be responsible for smoother occlusal surfaces, enabling self-cleaning with the tongue and facilitating oral hygiene. It has also been hypothesized that bruxism plays a protective role even in OSAS, although the evidence for this is not yet conclusive [62]. 

Apart from these possible positive consequences, bruxism may have several negative repercussions, which have been widely discussed in the literature. Bruxism has been associated with temporomandibular pain and dysfunction, induction of temporal tension headache, polygraphic observation of jaw muscle activity with audible teeth grinding sounds, masseter muscle hypertrophy, and facial pain [63,64]. Intraoral consequences include lip or cheek biting (worsened by the presence of xerostomia), tongue indentation, burning tongue with concomitant oral habits, periodontal and endodontic problems, failures of restorations and implants, abnormal tooth wear, and/or fractured teeth [63,64].

Bruxism itself does not require treatment, so an observational approach, such as symptoms monitoring, may be sufficient. Instead, management becomes mandatory when problems arise as a result of bruxism.

## 6. Management

There are multiple non-invasive methods for treating bruxism. They include oral appliances, biofeedback, the cognitive-behavioral approach, and the pharmacological approach.

Currently, there is no guideline for the treatment of bruxism in children and adolescents. The literature related to the management of bruxism in this population consists of a few case reports and clinical trials evaluating various interventions to reduce bruxism. This is partly justified by the fact that the majority of bruxist children do not maintain bruxism during adolescence and adulthood, thus preferring observational and non-interventional approaches in younger children [15]. Therefore, parents are encouraged to monitor any complaints their children may have and to have them evaluated at the dental office if they report pain when chewing or talking, or when they wake up [65].

Even more scarce is the literature inherent in the management of bruxism in DS children. In particular, only 4 studies, published between 1988 and 2020, investigated the treatment of bruxism in children and adolescents with DS. In 1988, Lamma and Cocchi published their pharmacological approach for the treatment of bruxism in children with DS. The authors reported interesting results. In 62.68% of cases the disorder was eliminated, and in a further 29.85% it was alleviated after 8–22 months of treatment [66]. That protocol was based on the idea of reducing the stress reactions to which the DS patient is subjected from the day of his conception [67]. Their study was performed on 134 children with DS and it included 9 treatment frameworks, to be as tailored as much as possible to each child’s needs. The researchers noted that already basic stress therapy (BAT; consisting of a combination of l-glutamine or I-glutamine + pemoline; pyridoxine or pyridoxine + thiamine or pyridoxine + thiamine + cyanocobalamine; sometimes the addition of alpha-tocopherol; a benzodiazepine) accounted for nearly a quarter of the cases treated. The addition of carbamazepine might be the best solution. Because this study is purely descriptive, the results can be taken only as an indication.

Aversive procedures have often been proposed for the treatment of repetitive behaviors in individuals with DS. On the contrary, Neil and Jones [68] investigated a method that employs the use of functional analysis procedures for determining the operant function of repetitive behavior and implements a differential reinforcement of other behavior (DRO) intervention in children with DS. In one of the 3 patients studied, the repetitive behavior, identified through the Repetitive Behavior Scale-Revised (RBS-R) [69], was AB as the target for assessment and treatment. The functional analysis conditions (attention, alone, demand, control, no-interaction, tangible) were alternated in a multi-element design. Each condition was presented once per day in a random order during 10-min-long sessions of functional analysis and treatment in which an observer recorded the occurrence of bruxism. Then, a reversal (ABAB) design was used to evaluate the effect of DRO on the frequency of bruxism. The Authors observed that the number of intervals in which bruxism occurred decreased dramatically when the DRO was introduced. The DRO presentation interval was gradually extended until it reached the baseline conditions; at this point, bruxism episodes increased and then decreased again with the re-introduction of DRO. Finally, the DRO presentation interval was faded to a level that maintained the reduction in the number of bruxism intervals over the subsequent 3 months of follow-up. The success obtained from this study reinforces the concept that repetitive behaviors can be reduced with positive procedures even in children with DS.

Regarding functional therapy in children with DS whose OSA falls within the mild range, positional treatments and mandibular advancement devices may be helpful, although these appliances have not been systematically evaluated in this population. These interventions are aimed at resolving airway obstructions in the nasopharynx, oropharynx, and hypopharynx, so both treatments for OSA and related SB would benefit. The problem with this approach is the lack of collaboration: providing an oral appliance in mentally impaired individuals is usually not successful. 

A protocol proposal for a randomized, controlled, blind, clinical trial for the treatment of SB in children with DS has been recently published [70]. Underlying this protocol, there are studies on acupuncture, a technique successfully used for the treatment of bruxism, obtaining a reduction in the activity of the masseter and anterior temporal muscles and an anxiety reduction as well [71]. Moreover, the protocol takes into consideration low-intensity laser therapy, since trigger point irradiation is an effective treatment for orofacial pain and reduction of swelling [72]. This study aims to analyze salivary levels of dopamine and cortisol and muscle activity before and after treatment with low-level laser therapy administered to acupoints in children with DS.

## 7. Conclusions

Relatively few studies have been performed on bruxism in children and adolescents with DS. A very high variability of results was observed, with a prevalence ranging from 18% to 79%, influenced by the different age groups studied and the different methods of assessing bruxism. This prevented the expression of a reliable estimate of its prevalence. Although a decreasing trend in this disorder has emerged as age increases (around 12 years), the lack of longitudinal studies does not allow us to predict the risk of bruxing even in adulthood. In conclusion, given the paucity of protocols for the management of bruxism in these children, it is hoped that new techniques will be tested in the future, as this is of paramount importance for all those cases in which an observational approach is not enough.

## Figures and Tables

**Table 1:** Published studies on bruxism in children with Down syndrome.

Author [Ref.]	n. of Patients	Age (Mean)	M/F Ratio	Diagnosis	Bruxism Prevalence
Alari et al. [24]	21	6–7 y	-	-	70%
Areias [37]	45	6–20 y (13)	1:0.96	Questionnaire + IO examination	23%
Ashworth [29]	22	6–12 y (9.42)	1:1	CSHQ, Actigraphy	45%
Bell [26]	49	4–18 y	1:1.13	IO and dental casts examination, IO photographs, diet diary, tooth wear and general health questionnaire	67% Tooth wear 11.4% SB * 8.6% AB *
Borea [28]	37	(12)	-	-	45%
Breslin [31]	35	7–18 y (12.65)	1:1.19	CSHQ	34% ^†^
Carter [30]	58	0.65–18 y (8.6)	1:1.07	CSHQ	45% (M: 55%, F: 33%) ^†^
Cocchi [25]	33	1–14 y (7.5)	1:1.54	-	60.61%
Gullikson [23]	28	3–10 y	-	-	78.8%
Hernández [35]	71	6–14 y	-	-	18%
López-Pérez [32]	57	3–15 y	1:1.48	Questionnaire, IO and dental casts examination	42% (M: 35%, F: 52%) 3–5.9 y: 29% 6–8.9 y: 59% 9–11.9 y: 50% 12–14.9 y: 10%
Maris [34]	54	5.4–11.6 y (7.5)	1:1.22	CSHQ	31.5% SB
Miamoto [36]	60	-	1:1.61	Questionnaire, IO examination	23% SB (M:24.3%, F:21.7%)
Ruy Carneiro [33]	112	5–16 y (8.4)	1:1.15	Questionnaire	51.8% (M:53.3%, F:50%) 5–7 y: 59.6% 8–12 y: 63.3% 13–16 y: 31.4%

M = males; F = females; CSHQ = Children’s Sleep Habits Questionnaire; IO = intra-oral; SB = sleep bruxism; AB = awake bruxism; y = years. * data based on 71.4% of the sample who filled the questionnaire. ^†^ Parasomnias (including bruxing and grinding) ≥ 2 nights/week.

## References

[1] Lobbezoo F., Ahlberg J., Glaros A.G., Kato T., Koyano K., Lavigne G.J., de Leeuw R., Manfredini D., Svensson P., Winocur E. (2013). Bruxism defined and graded: An international consensus. J. Oral Rehabil..

[2] Lobbezoo F., Ahlberg J., Raphael K.G., Wetselaar P., Glaros A.G., Kato T., Santiago V., Winocur E., De Laat A., De Leeuw R. (2018). International consensus on the assessment of bruxism: Report of a work in progress. J. Oral Rehabil..

[3] Guichet N.F. (1977). Occlusion: A Teaching Manual.

[4] Sateia M.J. (2014). International classification of sleep disorders-third edition: Highlights and modifications. Chest.

[5] Rugh J.D., Barghi N., Drago C.J. (1984). Experimental occlusal discrepancies and nocturnal bruxism. J. Prosthet Dent..

[6] Santarelli A., Mascitti M., Orsini G., Memè L., Rocchetti R., Tiriduzzi P., Sampalmieri F., Putignano A., Procaccini M., Lo Muzio L. (2014). Osteopontin, osteocalcin and OB-cadherin expression in Synthetic nanohydroxyapatite vs bovine hydroxyapatite cultured Osteoblastic-like cells. J. Biol. Regul. Homeost. Agents.

[7] Togni L., Mascitti M., Santarelli A., Contaldo M., Romano A., Serpico R., Rubini C. (2019). Unusual Conditions Impairing Saliva Secretion: Developmental Anomalies of Salivary Glands. Front. Physiol..

[8] Schiffman E.L., Fricton J.R., Haley D. (1992). The relationship of occlusion, parafunctional habits and recent life events to mandibular dysfunction in a non-patient population. J. Oral Rehabil..

[9] Ellison J.M., Stanziani P. (1993). SSRI-associated nocturnal bruxism in four patients. J. Clin. Psychiatry.

[10] Lindqvist B., Heijbel J. (1974). Bruxism in children with brain damage. Acta Odontol. Scand..

[11] Ohayon M.M., Li K.K., Guilleminault C. (2001). Risk factors for sleep bruxism in the general population. Chest.

[12] Lobbezoo F., Visscher C.M., Ahlberg J., Manfredini D. (2014). Bruxism and genetics: A review of the literature. J. Oral Rehabil..

[13] Bertazzo-Silveira E., Kruger C.M., Porto De Toledo I., Porporatti A.L., Dick B., Flores-Mir C., De Luca Canto G. (2016). Association between sleep bruxism and alcohol, caffeine, tobacco, and drug abuse: A systematic review. J. Am. Dent. Assoc..

[14] Machado E., Dal-Fabbro C., Cunali P.A., Kaizer O.B. (2014). Prevalence of sleep bruxism in children: A systematic review. Dent. Press J. Orthod..

[15] Manfredini D., Restrepo C., Diaz-Serrano K., Winocur E., Lobbezoo F. (2013). Prevalence of sleep bruxism in children: A systematic review of the literature. J. Oral Rehabil..

[16] Van Selms M.K., Visscher C.M., Naeije M., Lobbezoo F. (2013). Bruxism and associated factors among Dutch adolescents. Community Dent. Oral Epidemiol..

[17] Carra M.C., Huynh N., Morton P., Rompre P.H., Papadakis A., Remise C., Lavigne G.J. (2011). Prevalence and risk factors of sleep bruxism and wake-time tooth clenching in a 7- to 17-yr-old population. Eur. J. Oral Sci..

[18] Emodi Perlman A., Lobbezoo F., Zar A., Friedman Rubin P., van Selms M.K., Winocur E. (2016). Self-Reported bruxism and associated factors in Israeli adolescents. J. Oral Rehabil..

[19] Winocur E., Messer T., Eli I., Emodi-Perlman A., Kedem R., Reiter S., Friedman-Rubin P. (2019). Awake and Sleep Bruxism Among Israeli Adolescents. Front. Neurol..

[20] Kieser J.A., Groeneveld H.T. (1998). Relationship between juvenile bruxing and craniomandibular dysfunction. J. Oral Rehabil..

[21] De Graaf G., Buckley F., Skotko B.G. (2015). Estimates of the live births, natural losses, and elective terminations with Down syndrome in the United States. Am. J. Med. Genet. A.

[22] Dumortier L., Bricout V.A. (2020). Obstructive sleep apnea syndrome in adults with down syndrome: Causes and consequences. Is it a “chicken and egg” question?. Neurosci. Biobehav. Rev..

[23] Gullikson J.S. (1973). Oral findings in children with Down’s syndrome. Asdc. J. Dent. Child..

[24] Alarí B.M.E. (1994). The Infantile Bruxism.

[25] Cocchi R., Lamma A. (1999). Bruxismo e altri possibili sintomi di stress in bambini o giovani down e non-down, autistici o con altri disturbi generalizzati dello sviluppo. Ital. J. Intellective Impair..

[26] Bell E.J., Kaidonis J., Townsend G.C. (2002). Tooth wear in children with Down syndrome. Aust. Dent. J..

[27] Rodriguez Peinado N., Mourelle Martinez M.R., Dieguez Perez M., De Nova Garcia M.J. (2018). A study of the dental treatment needs of special patients: Cerebral paralysis and Down syndrome. Eur. J. Paediatr. Dent..

[28] Borea G., Magi M., Mingarelli R., Zamboni C. (1990). The oral cavity in Down syndrome. J. Pedod..

[29] Ashworth A., Hill C.M., Karmiloff-Smith A., Dimitriou D. (2013). Cross syndrome comparison of sleep problems in children with Down syndrome and Williams syndrome. Res. Dev. Disabil..

[30] Carter M., McCaughey E., Annaz D., Hill C.M. (2009). Sleep problems in a Down syndrome population. Arch. Dis. Child..

[31] Breslin J.H., Edgin J.O., Bootzin R.R., Goodwin J.L., Nadel L. (2011). Parental report of sleep problems in Down syndrome. J. Intellect Disabil. Res..

[32] Lopez-Perez R., Lopez-Morales P., Borges-Yanez S.A., Maupome G., Pares-Vidrio G. (2007). Prevalence of bruxism among Mexican children with Down syndrome. Downs Syndr. Res. Pract..

[33] Ruy Carneiro N.C., de Castro Souza I., Duda Deps Almeida T., Serra-Negra J.M.C., Almeida Pordeus I., Borges-Oliveira A.C. (2020). Risk factors associated with reported bruxism among children and adolescents with Down Syndrome. Cranio.

[34] Maris M., Verhulst S., Wojciechowski M., Van de Heyning P., Boudewyns A. (2016). Sleep problems and obstructive sleep apnea in children with down syndrome, an overwiew. Int. J. Pediatr. Otorhinolaryngol..

[35] Hernández P.J., Tello H.T., Ochoa R.G. (1998). Oral alterations in children with Down’s syndrome from Yucatán state. Rev. Adm..

[36] Miamoto C.B., Pereira L.J., Ramos-Jorge M.L., Marques L.S. (2011). Prevalence and predictive factors of sleep bruxism in children with and without cognitive impairment. Braz. Oral Res..

[37] Areias C.M., Sampaio-Maia B., Guimaraes H., Melo P., Andrade D. (2011). Caries in Portuguese children with Down syndrome. Clinics.

[38] Wetselaar P., Vermaire E.J.H., Lobbezoo F., Schuller A.A. (2021). The prevalence of awake bruxism and sleep bruxism in the Dutch adolescent population. J. Oral Rehabil..

[39] Owens J.A., Spirito A., McGuinn M. (2000). The Children’s Sleep Habits Questionnaire (CSHQ): Psychometric properties of a survey instrument for school-aged children. Sleep.

[40] Vanderas A.P., Manetas K.J. (1995). Relationship between malocclusion and bruxism in children and adolescents: A review. Pediatr. Dent..

[41] Carra M.C., Huynh N., Lavigne G. (2012). Sleep bruxism: A comprehensive overview for the dental clinician interested in sleep medicine. Dent. Clin. N. Am..

[42] Esbensen A.J., Hoffman E.K., Stansberry E., Shaffer R. (2018). Convergent validity of actigraphy with polysomnography and parent reports when measuring sleep in children with Down syndrome. J. Intellect Disabil. Res..

[43] Ancoli-Israel S., Cole R., Alessi C., Chambers M., Moorcroft W., Pollak C.P. (2003). The role of actigraphy in the study of sleep and circadian rhythms. Sleep.

[44] Sadeh A., Hauri P.J., Kripke D.F., Lavie P. (1995). The role of actigraphy in the evaluation of sleep disorders. Sleep.

[45] Macaluso G.M., Guerra P., Di Giovanni G., Boselli M., Parrino L., Terzano M.G. (1998). Sleep bruxism is a disorder related to periodic arousals during sleep. J. Dent. Res..

[46] Veatch O.J., Reynolds A., Katz T., Weiss S.K., Loh A., Wang L., Malow B.A. (2016). Sleep in Children With Autism Spectrum Disorders: How Are Measures of Parent Report and Actigraphy Related and Affected by Sleep Education?. Behav. Sleep Med..

[47] Ivanenko A., Gururaj B.R. (2009). Classification and epidemiology of sleep disorders. Child. Adolesc. Psychiatr. Clin. N Am..

[48] Kuhn M., Turp J.C. (2018). Risk factors for bruxism. Swiss Dent. J..

[49] Castroflorio T., Bargellini A., Rossini G., Cugliari G., Rainoldi A., Deregibus A. (2015). Risk factors related to sleep bruxism in children: A systematic literature review. Arch. Oral Biol..

[50] Lavigne G.J., Kato T., Kolta A., Sessle B.J. (2003). Neurobiological mechanisms involved in sleep bruxism. Crit. Rev. Oral Biol. Med..

[51] Negoro T., Briggs J., Plesh O., Nielsen I., McNeill C., Miller A.J. (1998). Bruxing patterns in children compared to intercuspal clenching and chewing as assessed with dental models, electromyography, and incisor jaw tracing: Preliminary study. Asdc. J. Dent. Child..

[52] Serra-Negra J.M., Ribeiro M.B., Prado I.M., Paiva S.M., Pordeus I.A. (2017). Association between possible sleep bruxism and sleep characteristics in children. Cranio.

[53] Oliveira A.C., Paiva S.M., Campos M.R., Czeresnia D. (2008). Factors associated with malocclusions in children and adolescents with Down syndrome. Am. J. Orthod Dentofac. Orthop..

[54] Guo H., Wang T., Niu X., Wang H., Yang W., Qiu J., Yang L. (2018). The risk factors related to bruxism in children: A systematic review and meta-analysis. Arch. Oral Biol..

[55] Lamenha Lins R.M., Cavalcanti Campelo M.C., Mello Figueiredo L., Vilela Heimer M., Dos Santos-Junior V.E. (2020). Probable Sleep Bruxism in Children and its Relationship with Harmful Oral Habits, Type of Crossbite and Oral Breathing. J. Clin. Pediatr. Dent..

[56] Oksenberg A., Arons E. (2002). Sleep bruxism related to obstructive sleep apnea: The effect of continuous positive airway pressure. Sleep Med..

[57] Royal College of Paediatrics and Child Health (2009). Working Party on Sleep Physiology and Respiratory Control Disorders in Childhood.

[58] Lumeng J.C., Chervin R.D. (2008). Epidemiology of pediatric obstructive sleep apnea. Proc. Am. Thorac. Soc..

[59] Rosen D. (2011). Management of obstructive sleep apnea associated with Down syndrome and other craniofacial dysmorphologies. Curr. Opin. Pulm. Med..

[60] Turner M. (1999). Annotation: Repetitive behaviour in autism: A review of psychological research. J. Child. Psychol. Psychiatry.

[61] Hollander E., Wang A.T., Braun A., Marsh L. (2009). Neurological considerations: Autism and Parkinson’s disease. Psychiatry Res..

[62] Manfredini D., Guarda-Nardini L., Marchese-Ragona R., Lobbezoo F. (2015). Theories on possible temporal relationships between sleep bruxism and obstructive sleep apnea events. An expert opinion. Sleep Breath.

[63] Okeson J.P., de Kanter R.J. (1996). Temporomandibular disorders in the medical practice. J. Fam. Pract..

[64] Lavigne G.J., Khoury S., Abe S., Yamaguchi T., Raphael K. (2008). Bruxism physiology and pathology: An overview for clinicians. J. Oral Rehabil..

[65] Okeson J.P. (2020). Management of Temporomandibular Disorders and Occlusion.

[66] Lamma A., Cocchi R. (1988). Drug Therapies of Bruxism in Down Children: Preliminary Report. Ital. J. Intellective Impair..

[67] Cocchi R., Lamma A. (1987). Bruxism in soggetti affetti da sindrome di Down. Studio epidemiologico su 366 casi. Odontostoinat. Implant..

[68] Neil N., Jones E.A. (2016). Repetitive behavior in children with Down syndrome: Functional analysis and intervention. J. Dev. Phys. Disabil..

[69] Bodfish J.W., Symons F.W., Lewis M.H. (1999). The Repetitive Behavior Scale-Revised.

[70] Salgueiro M., Silva T., Motta L.J., Horliana A., Goncalves M.L.L., Gomes A.O., Pinto M.M., Bortoletto C.C., Altavista O.M., Deana A.M. (2020). Effects of Photobiomodulation in Children with Down Syndrome and Possible Sleep Bruxism: Protocol For A Randomized, Controlled, Blind, Clinical Trial: Study protocol clinical trial (SPIRIT compliant). Medicine.

[71] Dallanora L.J., Faltin P.P., Inoue R.T., Santos V.M. (2004). Acupuncture use in the treatment of patients with bruxism. RGO.

[72] Venezian G.C., da Silva M.A., Mazzetto R.G., Mazzetto M.O. (2010). Low level laser effects on pain to palpation and electromyographic activity in TMD patients: A double-blind, randomized, placebo-controlled study. Cranio.

